# Genome‐wide SNP identification in *Fraxinus* linking genetic characteristics to tolerance of *Agrilus planipennis*

**DOI:** 10.1002/ece3.8163

**Published:** 2021-09-27

**Authors:** Cecelia E. Hale, Mark A. Jordan, Gloria Iriarte, Kirk Broders, Andrew J. Storer, Vamsi J. Nalam, Jordan M. Marshall

**Affiliations:** ^1^ Department of Biology Purdue University Fort Wayne Fort Wayne Indiana; ^2^ Bioagricultural Sciences and Pest Management Colorado State University Fort Collins Colorado; ^3^ Smithsonian Tropical Research Institute Panama City Panama; ^4^ College of Forest Resources and Environmental Science Michigan Technological University Houghton Michigan

**Keywords:** ash, emerald ash borer, RAD‐seq, survival, tolerance

## Abstract

Ash (*Fraxinus* spp.) is one of the most widely distributed tree genera in North America. Populations of ash in the United States and Canada have been decimated by the introduced pest *Agrilus planipennis* (Coleoptera: Buprestidae; emerald ash borer), having negative impacts on both forest ecosystems and economic interests. The majority of trees succumb to attack by *A. planipennis*, but some trees have been found to be tolerant to infestation despite years of exposure. Restriction site‐associated DNA (RAD) sequencing was used to sequence ash individuals, both tolerant and susceptible to *A. planipennis* attack, in order to identify single nucleotide polymorphism (SNP) patterns related to tolerance and health declines. de novo SNPs were called using SAMtools and, after filtering criteria were implemented, a set of 17,807 SNPs were generated. Principal component analysis (PCA) of SNPs aligned individual trees into clusters related to geography; however, five tolerant trees clustered together despite geographic location. A subset of 32 outlier SNPs identified within this group, as well as a subset of 17 SNPs identified based on vigor rating, are potential candidates for the selection of host tolerance. Understanding the mechanisms of host tolerance through genome‐wide association has the potential to restore populations with cultivars that are able to withstand *A. planipennis* infestation. This study was successful in using RAD‐sequencing in order to identify SNPs that could contribute to tolerance of *A. planipennis*. This was a first step toward uncovering the genetic basis for host tolerance to *A. planipennis*. Future studies are needed to identify the functionality of the loci where these SNPs occur and how they may be related to tolerance of *A. planipennis* attack.

## Graphical abstract

The majority of *Fraxinus* spp. individuals succumb to attack by *Agrilus planipennis* in North America, but some trees have been found to be tolerant to infestation despite years of exposure. Outlier SNPs (single nucleotide polymorphisms) were identified in a distinct cluster of *Fraxinus* individuals despite different geographic locations. This provides an initial step toward uncovering the genetic basis for host tolerance to *A. planipennis*.

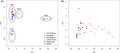

## 1 INTRODUCTION

*Agrilus planipennis* Fairmaire (Coleoptera: Buprestidae; emerald ash borer) is a metallic green beetle native to northeastern Asia that has become a pest to North American ash (*Fraxinus* spp. L.; McCullough et al., 2004). This pest was introduced into the Detroit/Windsor area of Michigan, USA/Ontario, Canada, and quickly dispersed via human assistance, including movement of firewood, nursery stock, and wood packing material (Buck & Marshall, 2008; Cappaert et al., 2005). In its native range, *A. planipennis* coevolved with Manchurian ash (*F*. *mandshurica* Rupr.) and is a secondary pest in this tree species, requiring a primary stressor for successful attack (Rebek et al., 2008; Whitehill et al., 2012). Kelly et al. (2020) confirmed that Asian ash species occur in distinct phylogenetic lineages with candidate genes for tolerance. Ash species in North America lack this natural resistance and succumb to attack, regardless of the presence of a primary stressor, often within one to four years after initial attack (Eyles et al., 2007; Rebek et al., 2008). While black (*F. nigra* Marsh.), green (*F. pennsylvanica* Marsh.), and white (*F. americana* L.) ash are the most susceptible in the introduced range of *A. planipennis*, all North American ash are susceptible (Anulewicz et al., 2006; Cappaert et al., 2005; Poland & McCullough, 2006). Blue ash (*F. quadrangulata* Michx.) has the lowest susceptibility to *A. planipennis* attack among North American ash species (Tanis & McCullough, 2012).

In this introduced range, the life cycle of *A. planipennis* is typically completed within one year (Herms & McCullough, 2014). Beginning in May, adults emerge through D‐shaped exit holes and mature within seven days as they feed on canopy leaves. Males identify suitable mates via visual and contact cues, and females feed on foliage for an additional five to seven days after mating before oviposition begins (Lelito et al., 2007, 2009; Poland & McCullough, 2006). During oviposition, females deposit eggs in bark cracks and crevices, where eggs hatch within two weeks, and larvae tunnel into the bark to feed on the phloem and vascular cambium of the tree from late summer to autumn. Phloem consumption creates serpentine‐shaped galleries, which severs photosynthate transport leading to eventual tree mortality. After completing four instars, larvae overwinter in pupal chambers and pupation occurs the following spring (Herms & McCullough, 2014; Poland & McCullough, 2006). While a one‐year life cycle is most common, a two‐year life cycle does occur, especially in more northern latitudes, with larvae overwintering in intermediate instars within the phloem (Wei et al., 2007).

The proliferation of *A. planipennis* throughout forests in North America has caused the mortality of millions of ash trees, producing devastating ecological and economic impacts (Flower et al., 2013; Kovacs et al., 2011; Poland & McCullough, 2006). These impacts have created long‐lasting changes to North American forest ecosystems and may require substantial restoration efforts (Herms & McCullough, 2014; Marshall, 2020; Poland & McCullough, 2006). Additional negative impacts include reduction in amount of wood products produced from ash and diminished esthetics in urban and suburban neighborhoods (Flower et al., 2013; Poland & McCullough, 2006). The cost of removal and replacement of ash trees in urban landscapes has been estimated at $12.5 billion from 2010 to 2020 (Kovacs et al., 2011). Additionally, the estimated loss by timberlands in the United States is $300 billion (Poland & McCullough, 2006).

Given the large‐scale distribution of *A. planipennis*, management options for control of the pest remain limited, which is further complicated by natural and urban landscapes. Therefore, a long‐term solution to preserving ash will depend on successfully identifying resistant or tolerant populations. Resistance to wood‐boring beetles is typically a function of female host selection and larval survival rate (Hanks, 1999). Therefore, resistance mechanisms can be placed into three general categories: antixenosis, antibiosis, and tolerance (Kogan & Ortman, 1978; Painter, 1951). Antixenosis traits are aimed at decreasing preferences for feeding and/or ovipositioning, while antibiosis results from traits that negatively affect insect growth, survival, and/or fecundity. Lastly, tolerance is the ability of the host to withstand infestation while remaining relatively healthy compared to other individuals undergoing the same level of attack.

There is evidence of antixenotic traits in the interaction between *A. planipennis* and hosts. Adults of *A. planipennis* express variation in both feeding and oviposition host preferences. When given a choice, adult beetles preferentially feed on white, green, and black ash compared with Manchurian, blue, and European ash (*F. excelsior* L.; Pureswaran & Poland, 2009). North American ash species receive more eggs compared with Manchurian ash, suggesting a female choice of susceptible hosts in order to increase larval performance (Gripenberg et al., 2010; Rigsby et al., 2014). Within North American ash species, inter‐ and intraspecific variation of volatile emissions and oviposition preferences of *A. planipennis* have been shown to play a role in resistance (Anulewicz et al., 2008; Chen et al., 2011; Koch et al., 2015). Bark of blue ash has a phenolic composition that may contribute to its resistance relative to white, green, and black ash (Whitehill et al., 2012). Bark smoothness, as a physical characteristic, may be a limiting factor in oviposition locations and subsequently limits the number of larvae that could attack a tree at a given time (Marshall et al., 2013). Additionally, variability in ash growth rates has been related to susceptibility to *A. planipennis*, with trees tolerant of attack having more rapid and constant growth compared to susceptible trees (Boyes et al., 2019).

Antibiosis interactions also exist in larval development. Mechanisms that affect larval performance mainly focus on variation in phenolic and defense protein chemistry (Cipollini et al., 2011; Villari et al., 2016; Whitehill et al., 2011, 2012). Previous studies comparing phenolic and lignin profiles of ash species found that Manchurian ash contains unique profiles that may contribute to their resistance to *A. planipennis* (Cipollini et al., 2011; Whitehill et al., 2011, 2012). Four potential defense‐related proteins are expressed more than fivefold higher in Manchurian ash than in other species and may contribute to resistance (Whitehill et al., 2011).

Mechanisms of tolerance are more difficult to quantify and therefore have not been as well studied. Identifying the genetic variants that allow these surviving trees in North America to tolerate infestation would greatly aide in the conservation of ash (Villari et al., 2016). Even with severe levels of ash mortality in the introduced range, certain trees have been able to survive after years of repeated exposure (Marshall et al., 2013). This has led to the identification of trees with differing apparent tolerance levels to *A. planipennis* attack. Trees classified as tolerant survive in spite of signs of *A. planipennis* attack and damage (Marshall et al., 2013). The objectives of this study were to (1) identify ash single nucleotide polymorphisms associated with the tolerance‐susceptibility gradient to *A. planipennis*, (2) identify phenotypic and genotypic relationships between trees relative to this tolerance‐susceptibility gradient, and (3) test the hypothesis that tolerance and susceptibility are linked to identifiable genetic markers.

## 2 METHODS

### 2.1 Study species and sample collection

**FIGURE 1:**
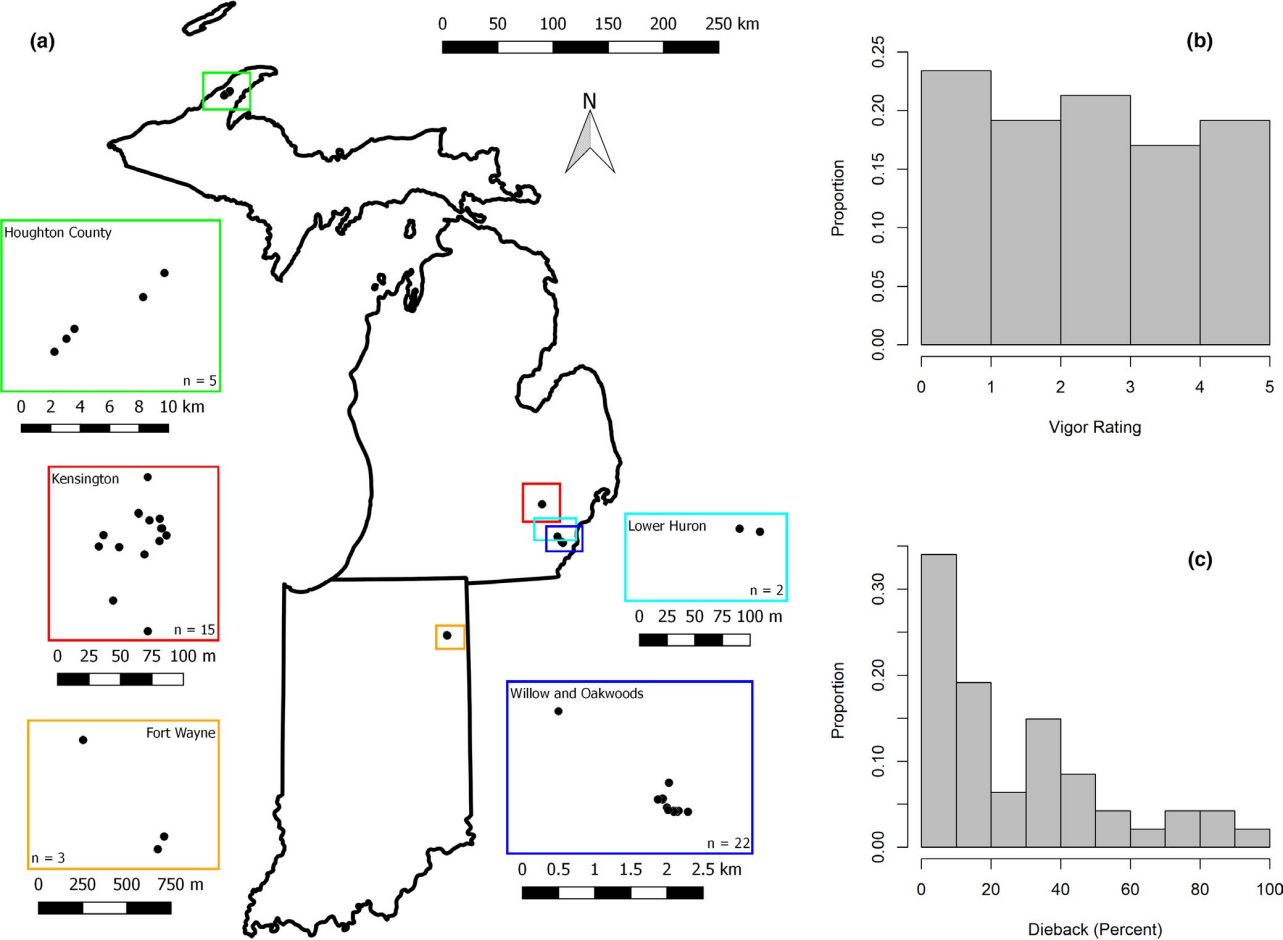
Location of sampled trees in Fort Wayne, Indiana, USA (a); Huron‐Clinton Metroparks, Michigan, USA (Kensington, Lower Huron, Oakwoods, and Willow); and Houghton County, Michigan, USA. Proportion of trees per category of vigor (b) and dieback (c) determined by field assessments

Trees were selected from *Fraxinus* spp. individuals within Fort Wayne, Indiana, USA (*n* = 3); Huron‐Clinton Metroparks, Michigan, USA (Kensington, Lower Huron, Oakwoods, and Willow; *n* = 39); and Houghton County, Michigan, USA (*n* = 5; Figure 1). All individuals were naturally occurring and not planted. Within most of these locations, green ash was the dominant species with white ash being less common. However, in Houghton County, white ash dominated. Selection of trees was based on their occurrence along an apparent conceptual gradient from high tolerance to high susceptibility (i.e., low tolerance) to *A. planipennis* attack, similar to a gradient described by Hietala (2013). Apical buds were collected from trees and placed in liquid nitrogen immediately after collection. Fort Wayne and Huron‐Clinton Metropark collections were made in July 2014. Houghton County collections were made in August 2016. Once returned to the laboratory, samples were stored at −80℃.

### 2.2 Tree assessment

Selected trees were identified to species and assessed on vigor (overall tree health: categorical 1–5 with 1 being high vigor [crown with relatively few dead twigs; normal foliage color and density] and 5 being low vigor [more than half of crown dead]), crown dieback (percent of dead branch tips: 5%–100%), and signs of *A. planipennis* attack (presence/absence: bark splits, exit holes, woodpecker damage, epicormic sprouts). Assessments followed those conducted in previous studies (Clark et al., 2015; Marshall et al., 2009, 2010, 2012, 2013), which were derived from Millers et al. (1991). After assessment, 47 individuals were selected for analysis and given an overall categorization of tolerant or susceptible to *A. planipennis* infestation. This tolerant‐susceptible categorization was similar to Hietala (2013). Individuals with a vigor ≤3 and dieback of ≤30% were considered tolerant. Individuals with a vigor of ≥3 and dieback >30% were considered susceptible. Chi‐square analysis was used to test the null hypothesis that tolerance categorization was independent of species. Additionally, diameter at breast height (dbh, 1.37 m above soil surface) was collected as a size metric and compared between species and tolerance groups using a two‐way analysis of variance (ANOVA).

### 2.3 DNA extraction and quantification

Entire bud samples (two to three buds) were homogenized using sterile ceramic mortars and pestles, which were first cooled with liquid nitrogen. DNeasy Plant Mini Kit (QIAGEN) was used to extract total genomic DNA following the manufacturer's protocol. DNA from each sample was quantified using UV spectrophotometry (NanoDrop 1000) absorbance. All samples were subsequently diluted to a concentration of 25 ng/μl.

### 2.4 Library creation and SNP discovery

Genomic DNA was converted into nextRAD genotyping‐by‐sequencing libraries (SNPsaurus, LLC) as described by Russello et al. (2015). Briefly, genomic DNA was first fragmented with Nextera reagent (Illumina, Inc.), which also ligates short adapter sequences to the ends of the fragments. The Nextera reaction was scaled for fragmenting 7 ng of genomic DNA, although 14 ng of genomic DNA was used for input to compensate for the amount of degraded DNA in the samples and to increase fragment sizes. Fragmented DNA was then amplified for 25 cycles at 75℃, with one of the primers matching the adapter and extending eight nucleotides into the genomic DNA with the selective sequence TGCAGGAG. Thus, only fragments starting with a sequence that can be hybridized by the selective sequence of the primer were efficiently amplified. The nextRAD libraries were sequenced on a HiSeq 4000 with one lane of 150 bp reads (University of Oregon).

The genotyping analysis used custom scripts (SNPsaurus, LLC) that trimmed the reads using bbduk (BBMap tools, http://sourceforge.net/projects/bbmap/). Command was as follows:





bash bbmap/bbduk.sh in=$file out=$outfile ktrim=r k=17 hdist=1 mink=8 ref=bbmap/resources/nextera.fa.gz minlen=100 ow=t qtrim=r trimq=10








Next, a de novo reference genome was created by collecting 10 million reads in total, evenly from the samples, and excluding reads that had counts fewer than 7 or more than 700. The remaining loci were then aligned to each other to identify allelic loci and collapse allelic haplotypes to a single representative. All reads were mapped to the reference with an alignment identity threshold of 95% using bbmap (BBMap tools). In order to assess the proportion of sequence reads that originated from *Fraxinus* spp. versus other species, 1,000 high‐quality reads from each sample were subject to BLASTN analysis in the NCBI database.

Genotype calling was done using SAMtools and BCFtools (Li et al., 2009). Command was as follows:





samtools mpileup ‐gu ‐Q 12 ‐t DP, DPR ‐f ref.fasta ‐b samples.txt | bcftools call ‐cv ‐ > genotypes.vcf






The VCF file was filtered to remove alleles with a population frequency of less than 0.03. Loci were removed that were heterozygous in all samples or had more than two alleles in a sample (suggesting collapsed paralogs). The absence of artifacts was checked by counting single nucleotide polymorphisms (SNPs) at each read nucleotide position and determining that SNP number did not increase with reduced base quality at the end of the read. All polymorphic sequences retained were subject to BLASTN analysis in the NCBI database.

VCFtools (Danecek et al., 2011) was used to further filter SNPs based on the following criteria: (1) Phred quality score, (2) minor allele frequency, (3) maximum missing genotype, and (4) minimum mean read depth (Table 1). Loci that failed to meet the quantification threshold for any of the filtering criteria were removed and excluded from subsequent analyses. Samples were not filtered based on Hardy–Weinberg expectations because the goal of this study was to identify polymorphic loci under selection, which are expected to deviate from equilibrium. The VCF file was converted into file formats necessary for analysis using PGDSpider 2.1.1.3 (Lischer & Excoffier, 2012).

**TABLE 1:** Filtering criteria for polymorphic loci

Filtering Criteria	Quantification
Heterozygous in all samples	False
More than 2 alleles in a sample	False
Phred‐like quality score	>20
Minor allele frequency	>0.1
Maximum missing genotype	0.5
Minimum mean read depth	14

Note

Loci failing to meet the quantification threshold for any of the criteria were excluded from subsequent analysis.

### 2.5 SNP variation

PLINK v 1.07 (Purcell et al., 2007) was used to assess stratification by phenotype within the filtered SNP data. Initially, phenotypes were set as coded species (green ash = 1 [control], white ash = 2 [case]). Using a permutation test of pairwise identity‐by‐state (IBS) distance, we tested the hypothesis that green and white ash individuals were less similar between than within putative species. Phenotypes were then coded as tolerance groups (tolerant = 1 [control], susceptible = 2 [case]). The pairwise permutation test of IBS was used to test the hypothesis that tolerant and susceptible individuals were less similar. Pairwise IBS distance tests were run with default 100,000 permutations. All PLINK functions included the ‐‐allow‐no‐sex option as both green and white ash are dioecious.

The packages *vcfR* v1.7.0 and *adegenet* v2.1.1 in R v3.4.2 (Jombart, 2008; Knaus & Grünwald, 2017; R Core Team, 2017) were used to perform an individual‐based principal component analysis (PCA) to characterize structure based on SNP variation. PCA was also used to visualize relationships between individuals based on phenotypic characteristics (vigor, dieback, and signs of *A. planipennis* attack) using *prcomp* R base function. Pearson's correlation was used to test the hypothesis that the first principal component axis for SNP PCA and the first principal component axis for phenotypic PCA had a linear relationship. Similarly, linear correlations were tested between the first principal component axis for SNP PCA with vigor and dieback values.

### 2.6 Detection of markers under selection

BAYESCAN v2.1 (Foll & Gaggiotti, 2008) was used to identify outlier loci based on populations defined by the PCA clusters, as well as populations defined by vigor rating. BAYESCAN uses a hierarchical Bayesian method to estimate population‐specific *F_ST_* coefficients as a fixation index, described by Beaumont and Balding (2004). A more conservative neutral model available in BAYESCAN (prior odds = 1,000) was used to minimize the number of false positives. Prior odds or prior probability is the likelihood of the null hypothesis being true before the test is performed. This increase in prior odds corresponds to the selection model being 1,000 times less likely than the neutral model, which was a more appropriate assumption given the number of SNPs included in this analysis (Lotterhos & Whitlock, 2014). After 100,000 iterations, SNPs with a posterior distribution over 0.95 were considered outliers. High *F_ST_* values (outliers) suggest that the locus has undergone directional selection (in contrast to balancing selection). Fisher's exact test was used in PLINK v1.07 (Purcell et al., 2007) to test the association of above identified outliers with the tolerance groups.

## 3 RESULTS

### 3.1 Phenotypic classification

The 47 ash trees selected for this study were sampled from six different geographic locations (Figure 1a). Based on morphological characteristics, nine trees were identified as white ash and the remaining trees were identified as green ash. The trees were classified into two major groups: tolerant or susceptible to *A. planipennis* attack based on both vigor and dieback ratings. This categorization resulted in 28 trees being classified as tolerant and 19 as susceptible. Vigor rating categories were evenly represented within each category (Figure 1b). However, dieback was skewed right, with fewer trees having greater values of dieback (Figure 1c). Overall, signs of *A. planipennis* infestation (i.e., bark splits, exit holes, woodpecker damage, epicormic sprouts) were present in 37 individuals, 31 of which had more than one sign present. Of the ten trees lacking signs, eight were categorized as tolerant and two were categorized as susceptible. There was a significant difference in dbh between species (*F* = 6.19, *df* = 1,43, *p*‐value = .017) with white being larger than green. However, there was no difference in dbh among tolerance groups (*F* = 0.40, *df* = 1,43, *p*‐value = .531) and the interaction between species and tolerance groups was not significant (*F* = 0.58, *df* = 1,43, *p*‐value = .449).

### 3.2 RAD‐sequencing

Restriction site‐associated DNA sequencing was used to sequence the genomes of 47 ash trees in order to identify SNPs that are correlated with tolerant or susceptible phenotypes. BLASTN analysis of the 1,000 random sequences from each individual revealed that 60.7% of the genomic sequences were potentially ash‐specific (no hits discovered). A high percentage of mitochondrial reads (18.0%) was found, potentially diverting reads from nuclear loci. Of sequences mapped, 17.7% aligned to *Populus tremula*, 0.5% *Sesamum* spp., and 0.5% *Olea europaea*. Due to the high mitochondrial hit counts, a reference of just polymorphic loci was created and BLASTN analysis was performed on those sequences. Out of this entire subset, only eight loci mapped to mitochondrial sequences.

**FIGURE 2:**
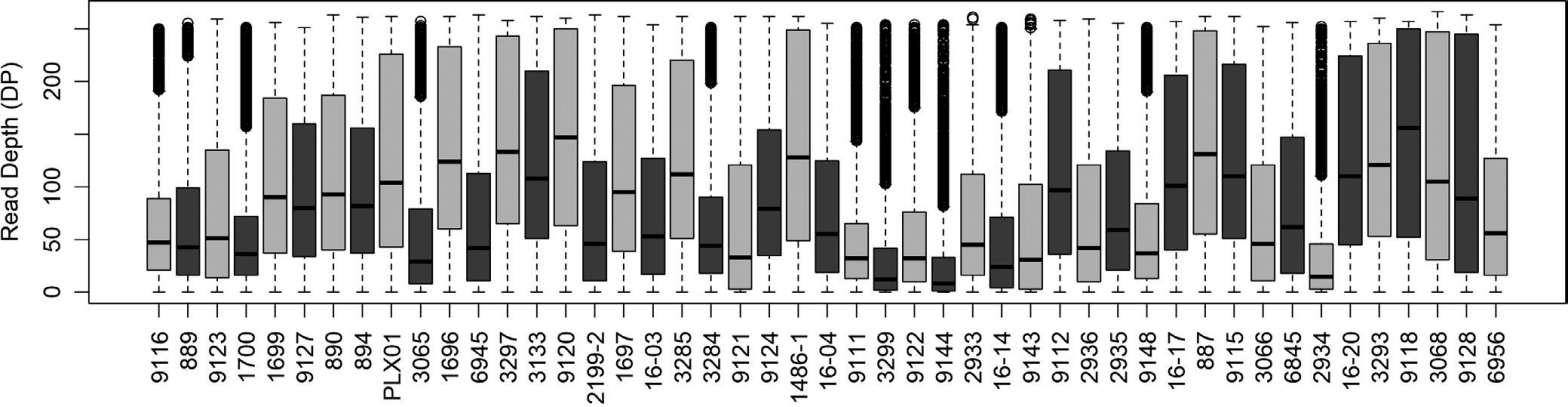
Boxplot of read depth for all sampled ash individuals

After SNP calling and initial filtering, a set of 23,243 SNPs were produced. Application of the more stringent filtering criteria (Table 1) generated a final set of 17,807 SNPs. Individuals were assessed for read depth. Mean read depth over all individuals was 91.5, and means ranged from 30 to 150 (Figure 2).

### 3.3 SNP variation

Pairwise IBS distance test to compare SNP similarity between species (green and white) failed to reject the hypothesis that pairs between the two species were less similar than pairs within species (*p*‐value = .240). This lack of variability between pairs extended into the other pairwise tests, with green pairs not more similar than white pairs (*p*‐value = .756). Additionally, the pairs between tolerance groups (tolerant and susceptible) were not less similar than pairs within groups (*p*‐value = .620). However, susceptible pairs were more similar than tolerant pairs (*p*‐value = .027).

**FIGURE 3:**
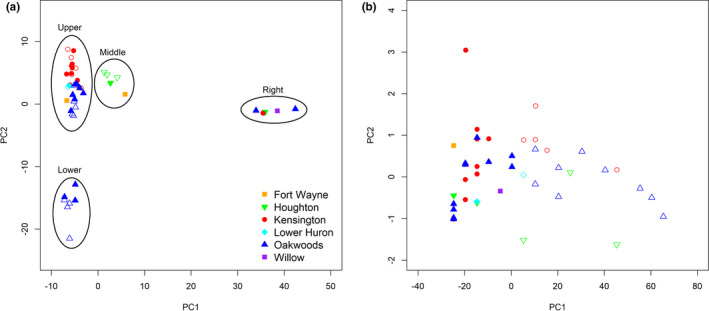
Principal component analysis (PCA) of filtered single nucleotide polymorphisms (SNPs) (a) and phenotypic data (b) from ash individuals in Fort Wayne, Indiana, USA; Huron‐Clinton Metroparks, Michigan, USA (Kensington, Lower Huron, Oakwoods, and Willow); and Houghton County, Michigan, USA. Closed symbols represent tolerant trees and open symbols represent susceptible trees. Ellipses are arbitrary, represent visual clustering, and are presented for labeling of cluster locations in PCA

Four major clusters (labeled as right, lower, upper, and middle) were identified in the PCA based on 17,807 SNPs (Figure 3a). Diverging substantially from all other groups, the cluster on the right contained trees from different geographic locations, including Houghton and three Metroparks (Kensington, Oakwoods, and Willow). Furthermore, all the individuals in this cluster were classified as tolerant to *A. planipennis* attack (Figure 3a). IBS clustering resulted in a multidimensional scaling ordination plot that did not differ from the visual clusters present in the PCA ordination plot.

### 3.4 Outlier SNPs

**FIGURE 4:**
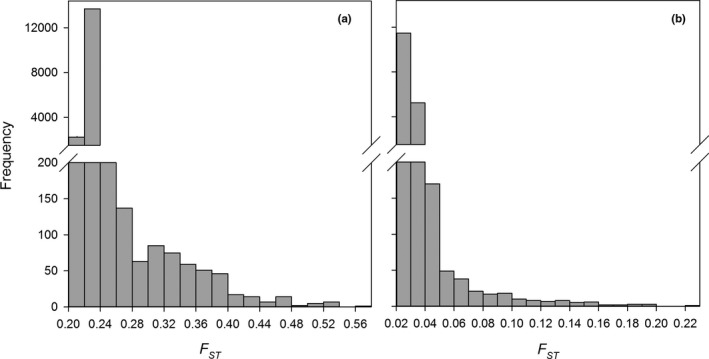
Frequency of *F*_ST_ values of all SNPs computed between populations based on the PCA clusters (a) and populations based on vigor rating (b)

Outlier detection based on the four PCA clusters identified 32 outlier SNPs within 28 different loci (Table 2). Of these outliers detected with the PCA clusters, nine were significantly associated with the tolerant group. Outlier detection based on vigor rating identified 17 outlier SNPs with 13 different loci (Table 3). Of these outliers detected with vigor rating, five were significantly associated with the tolerant group and five were significantly associated with the susceptible group (Table 3). All outlier *F_ST_* values were skewed to the right and outliers were relatively high, suggesting directional selection (Figure 4). There were no outliers detected by analysis when trees were grouped based on tolerance and susceptibility. Out of all 41 outlier loci, only one matched to a known sequence from the NCBI nucleotide database (locus 30133_137; top blast hit: XM_011468015.1). This locus mapped to a PTI1‐like tyrosine‐protein kinase receptor.

**TABLE 2:** Summary of outlier loci detected between four clusters within PCA

Locus	SNP Location	SNP	*F_ST_*	*q* Value	Tolerant	Susceptible	*p*‐Value
2690_69	86	G/A	0.472	0.045	0.21	0.00	.003
3535_59	67	A/G	0.478	0.024	0.21	0.09	.150
6512_10	66	A/G	0.472	0.042	0.22	0.03	.012
8409_8	142	A/G	0.533	0.002	0.25	0.38	.314
9230_115	99	C/G	0.479	0.029	0.18	0.09	.345
12476_19	13	C/A	0.466	0.019	0.29	0.06	.016
13284_148	49	G/A	0.504	0.031	0.15	0.07	.470
13980_8	103	T/C	0.505	0.006	0.17	0.03	.043
16669_22	10	T/C	0.532	0.004	0.26	0.12	.145
16669_22	13	A/G	0.532	0.003	0.26	0.12	.145
16669_22	49	T/C	0.531	0.004	0.26	0.12	.145
16669_22	104	T/C	0.527	0.004	0.26	0.12	.145
17461_7	34	C/T	0.441	0.047	0.27	0.14	.191
18944_8	106	T/C	0.437	0.05	0.23	0.22	1.000
22135_11	3	T/A	0.476	0.008	0.29	0.11	.042
24473_11	46	T/A	0.505	0.002	0.33	0.08	.005
30133_137	109	G/A	0.486	0.014	0.22	0.06	.039
31830_23	118	C/T	0.479	0.026	0.27	0.00	.001
34843_20	109	G/A	0.465	0.007	0.45	0.50	.806
37961_17	133	C/T	0.531	0.001	0.48	0.34	.263
38762_25	57	G/A	0.474	0.035	0.54	0.33	.102
39733_10	132	G/A	0.478	0.041	0.21	0.03	.024
50806_23	97	C/G	0.563	0.0002	0.40	0.19	.060
51756_10	107	A/C	0.469	0.011	0.23	0.24	1.000
56570_57	87	T/A	0.474	0.039	0.13	0.11	1.000
56570_57	132	C/A	0.471	0.037	0.17	0.11	.536
56747_9	86	C/T	0.441	0.033	0.19	0.18	1.000
57187_42	84	G/A	0.536	0.003	0.40	0.19	.054
59640_10	101	G/A	0.506	0.005	0.28	0.13	.151
61718_43	96	T/C	0.485	0.022	0.17	0.06	.284
67007_71	5	C/A	0.461	0.017	0.40	0.34	.636
88261_24	115	T/C	0.51	0.004	0.26	0.13	.253

Note

Includes single nucleotide polymorphism (SNP) location within loci, reference/polymorphic nucleotide pairs, and *F_ST_* and *q* Values. False discovery rate of <0.05 was used. Frequency in tolerant and susceptible groups from association Fisher's exact test, with *p*‐value.

**TABLE 3:** Summary of outlier loci detected between populations based on vigor rating

Locus	SNP Location	SNP	*F_ST_*	*q* Value	Tolerant	Susceptible	*p*‐Value
4467_128	20	A/G	0.190	0.004	0.35	0.04	.003
4467_128	28	T/C	0.199	0.002	0.39	0.04	.001
4467_128	55	A/G	0.192	0.005	0.35	0.04	.003
10225_13	51	C/T	0.163	0.047	0.35	0.03	<.001
19593_14	42	T/A	0.174	0.029	0.04	0.29	.001
25780_8	16	T/C	0.186	0.019	0.41	0.22	.097
39536_56	126	C/T	0.237	0.001	0.13	0.47	.002
41669_24	113	T/C	0.157	0.033	0.12	0.40	.010
46716_44	9	C/T	0.204	0.012	0.13	0.00	.056
46716_44	11	G/A	0.206	0.015	0.13	0.00	.056
49707_11	44	A/G	0.209	.003	0.39	0.30	.470
50319_14	27	C/T	0.16	0.037	0.41	0.20	.080
50319_14	102	C/T	0.166	0.026	0.37	0.13	.035
56942_24	101	C/T	0.179	0.009	0.27	0.53	.034
58136_88	134	A/G	0.194	0.017	0.43	0.53	.511
71869_12	51	G/A	0.162	0.042	0.24	0.38	.219
90283_36	123	C/T	0.157	0.022	0.23	0.72	<.001

Note

Includes single nucleotide polymorphism (SNP) location within loci, reference/polymorphic nucleotide pairs, and *F_ST_* and *q* Values. False discovery rate of <0.05 was used. Frequency in tolerant and susceptible groups from association Fisher's exact test, with *p*‐value.

**FIGURE 5:**
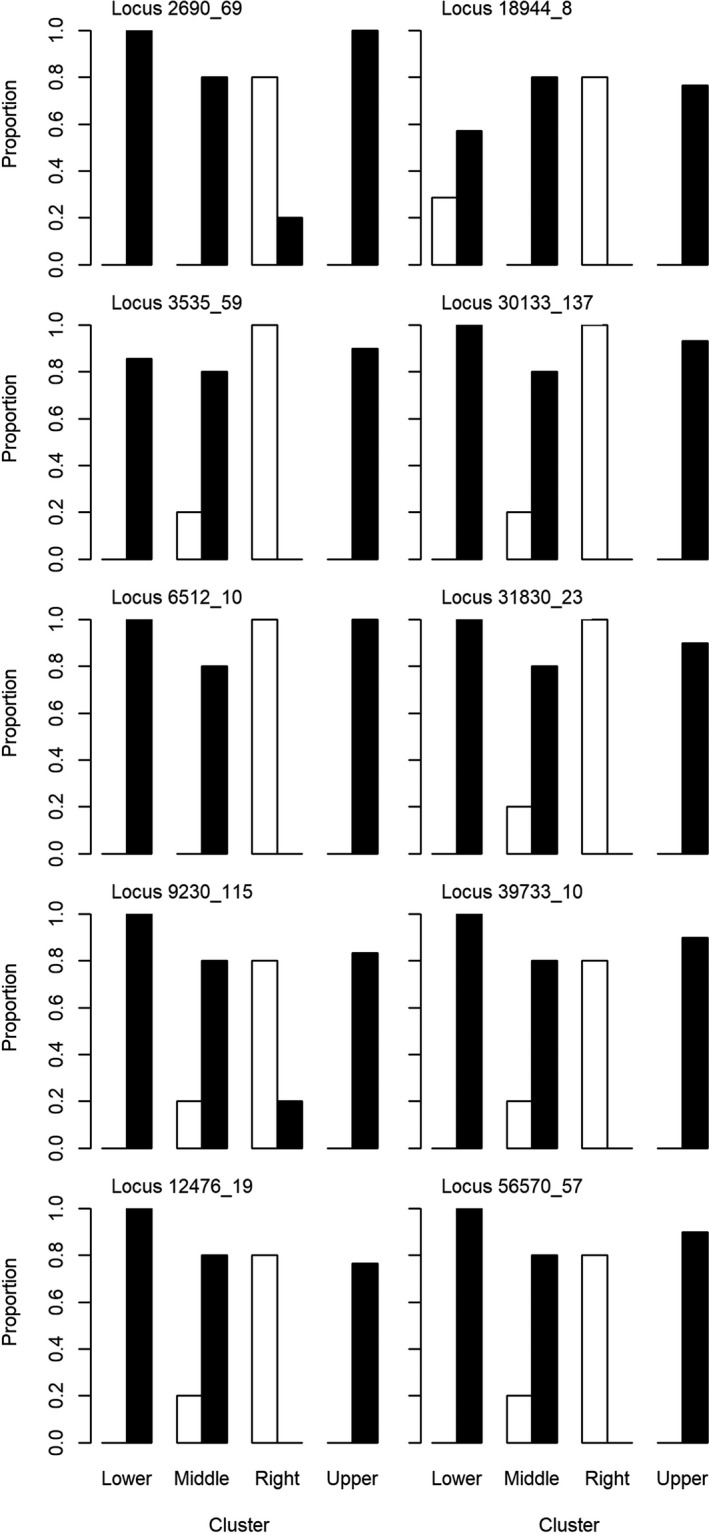
Proportion of trees in each PCA cluster that had the polymorphic nucleotide (white bars) or the reference nucleotide (black bars) for the ten outlier loci identified as having a clear presence in the right group. Within each locus, there was only one outlier SNP identified. Sample sizes for each cluster were as follows: right (*n* = 5), lower (*n* = 7), upper (*n* = 30), middle (*n* = 5). Samples with missing data for that loci were not included

**FIGURE 6:**
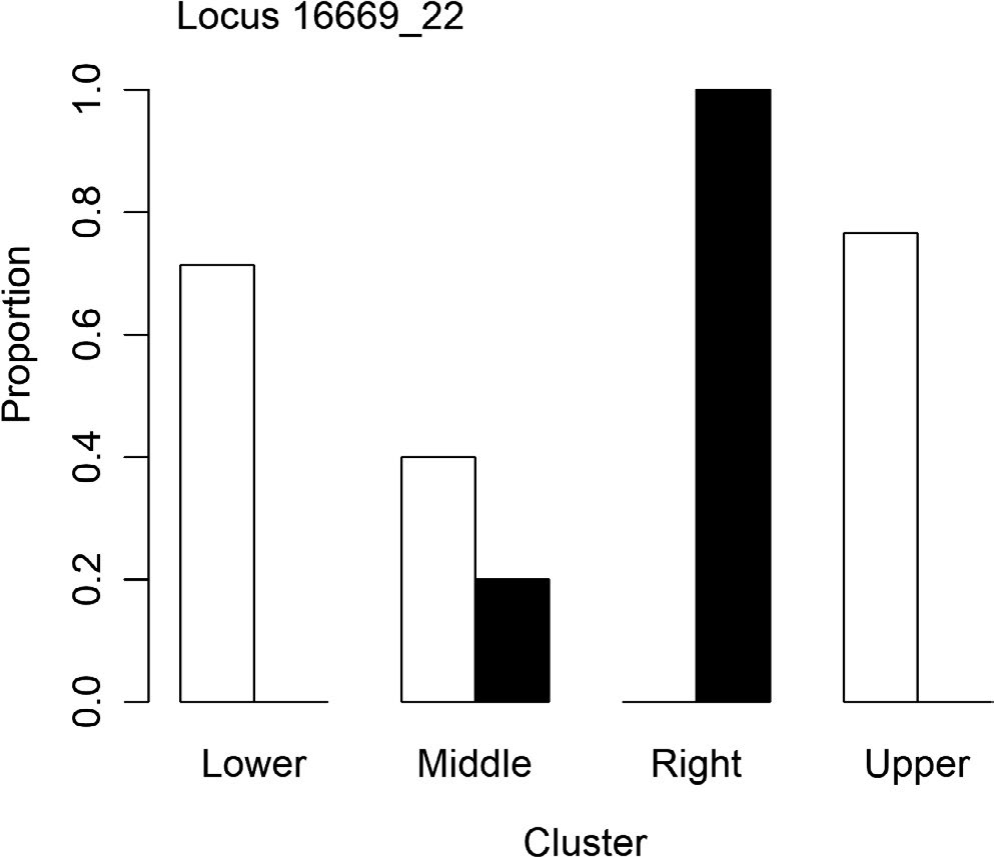
Proportion of trees in each PCA cluster that had the polymorphic nucleotide (white bars) or the reference nucleotide (black bars) for the locus 16669_22. Four SNPs were identified as outliers within this one locus (Table 3). Sample sizes for each cluster were as follows: right (*n* = 5), lower (*n* = 7), upper (*n* = 30), middle (*n* = 5). Samples with missing data were not included

Of the outliers detected between the PCA clusters, ten had a clear pattern of the polymorphic nucleotide being predominantly present in the five right cluster individuals (Figure 5). These patterns were slightly offset by similar genetic trends between the middle and right clusters; however, the right cluster clearly had the highest occurrence of these polymorphisms. Interestingly, one set of outlier SNPs, all occurring at the locus 16669_22, was present in all trees except the five in the right group and two trees from the middle group (Figure 6). These trees retained the reference nucleotide in this case, not the polymorphic nucleotide. For the outlier SNPs present at this locus, each individual either had all four polymorphic nucleotides or retained all four reference nucleotides.

**FIGURE 7:**
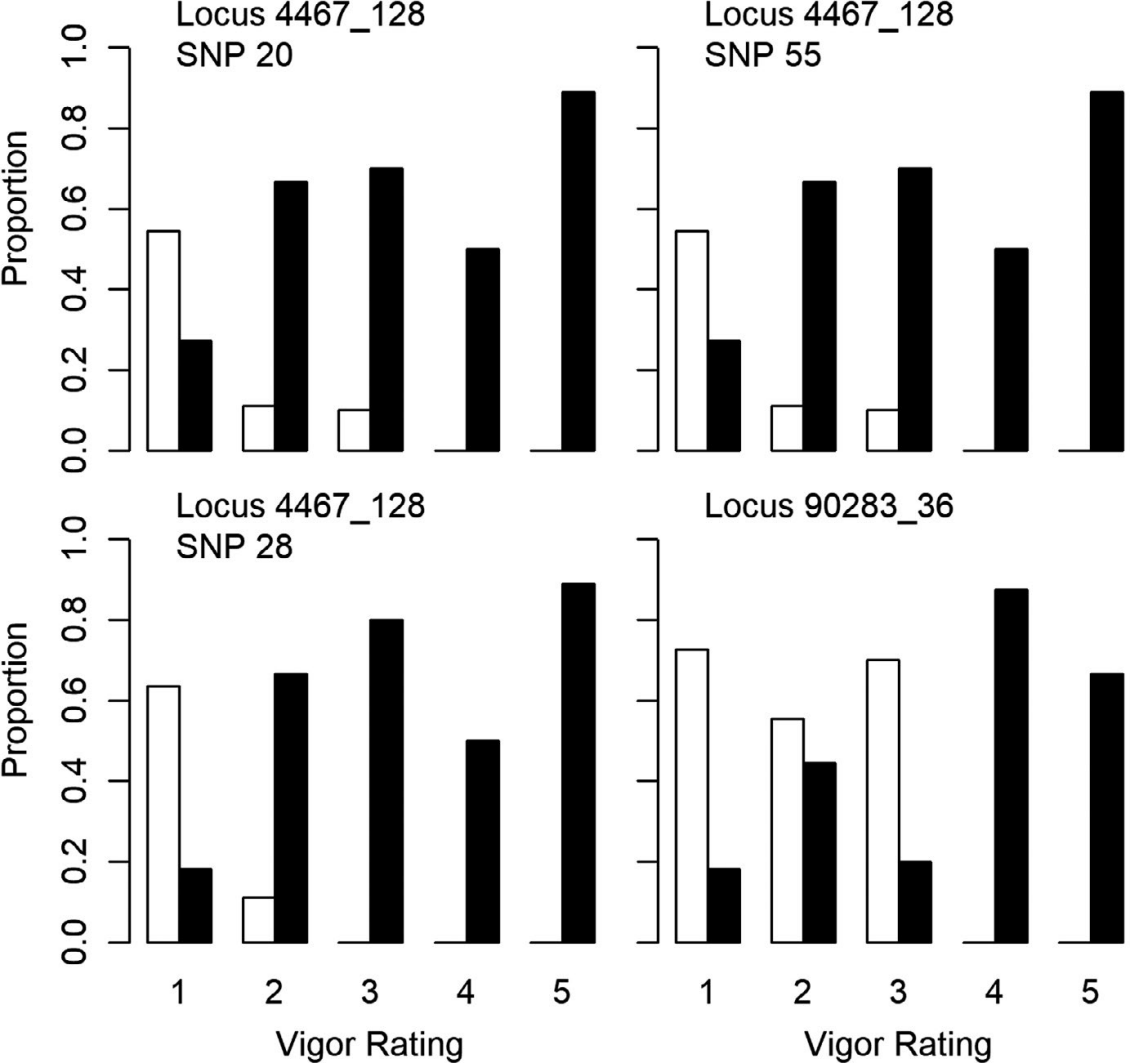
Proportion of trees in each vigor rating that had the polymorphic nucleotide (white bars) or the reference nucleotide (black bars) for the four outlier loci identified as having a clear pattern of presence in the high vigor groups. Sample sizes for each group were as follows: 1 (*n* = 11), 2 (*n* = 9), 3 (*n* = 10), 4 (*n* = 8), 5 (*n* = 9). Samples with missing data were not included

**FIGURE 8:**
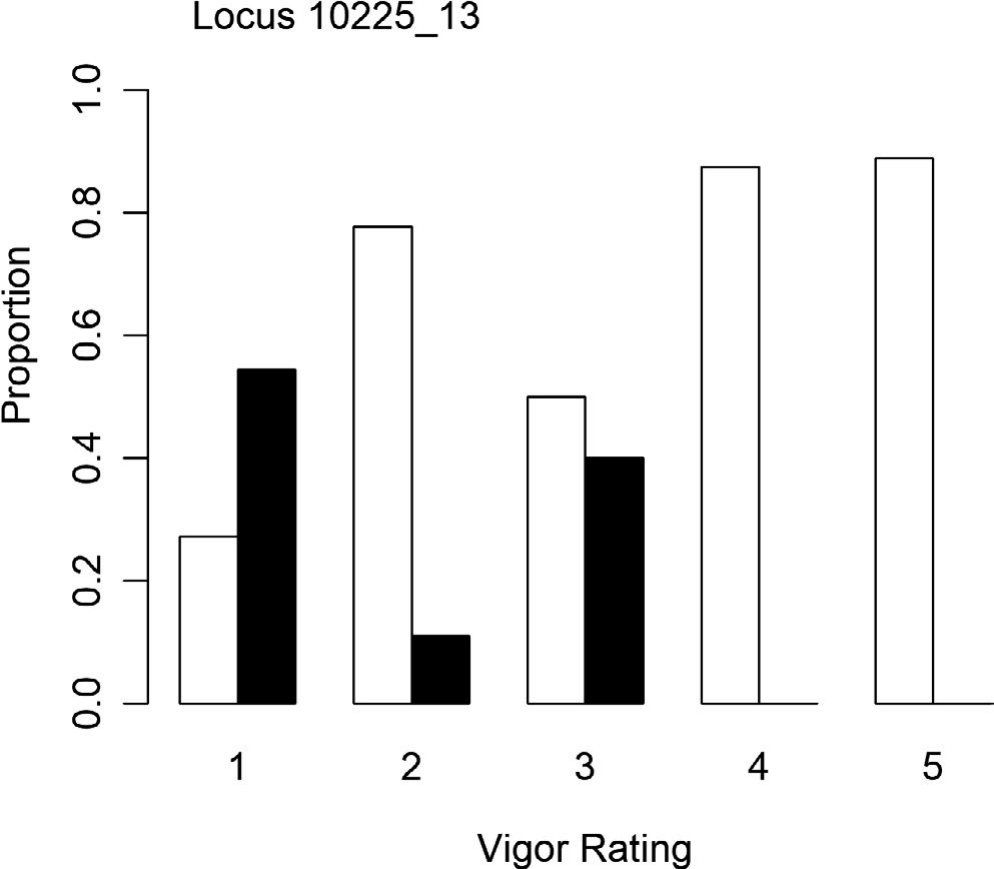
Proportion of trees in each vigor rating that had the polymorphic nucleotide (white bars) or the reference nucleotide (black bars) for the locus 10225_13. Sample sizes for each cluster were as follows: 1 (*n* = 11), 2 (*n* = 9), 3 (*n* = 10), 4 (*n* = 8), 5 (*n* = 9). Samples with missing data were not included

Of the outliers detected between groups based on vigor rating, four had a clear pattern of the polymorphic nucleotide being present exclusively in trees with high vigor (Figure 7). Three of these SNPs were present at one locus (4467_128). One outlier SNP at locus 10225_13 displayed a pattern of the reference nucleotide occurring more frequently in trees with high vigor (i.e., low vigor rating values), whereas trees with low vigor (i.e., high vigor rating values) all had the polymorphic nucleotide (Figure 8).

### 3.5 Phenotypic analysis

PCA based on all phenotypic data resulted in no distinct clustering between geographic locations (Figure 3b). Tolerant and susceptible trees did separate in this PCA; however, this is due to the categorization being defined by the same phenotypic data used to calculate the PCA. The PC1 axes of both the SNP and phenotype PCA analyses were not correlated (*r* = −0.16, *p*‐value = .270). There was no correlation between the SNP PC1 axis and vigor (r = −0.22, *p*‐value = .132). Likewise, there was no correlation between the SNP PC1 axis and dieback (*r* = −0.16, *p*‐value = .271).

## 4 DISCUSSION

This study identified polymorphic loci in *Fraxinus* spp. using RAD‐sequencing genotyping‐by‐sequencing. The filter settings were selected to ensured high‐quality nucleotide data with sufficient coverage across individuals (Bao et al., 2014; Nielsen et al., 2011). The resulting SNPs were used to highlight insights into a potential genetic basis for host tolerance to *A. planipennis*.

While the two species were different in regard to mean dbh, there no differences between the two tolerance groups. Size is an important factor in *A. planipennis* infestation, with larger trees (≥25 cm dbh) being the most likely attacked (Marshall et al., 2009). However, size importance in subsequent mortality is not as strong, as other physiological factors (e.g., increased bark roughness, decreased growth rates) may be more important in mortality (Boyes et al., 2019; Marshall et al., 2013).

### 4.1 Patterns of genetic variation

While the distance pairwise tests did not identify tolerance group pairs as more similar than across the two groups, there was less variation in the susceptible group pairs compared to the tolerant group pairs. PCA plots provided visual representation of genetic divergence among individuals. The PCA adds to the quantitative pairwise analysis in that visual clusters contained both tolerant and susceptible individuals, except for the cluster labeled “Right.” There was no clear relationship between the PCA clusters and geographic distribution, as three of the four clusters contained trees from multiple locations.

Field identified green and white ash did not separate as expected either—qualitatively in the PCA or quantitatively in pairwise distance analysis. This is not the first occurrence of genetic overlap of these two species. White ash is a polyploid species (2n = 46, 92, and 138) but most often diploid similar to green ash, and hybridization appears to confound genetic results (Schlesinger, 1990; Wallander, 2008; Whittemore et al., 2018). In some cases, white ash individuals group with green ash in phylogenetic analyses, a result that was attributed to the white ash samples likely being a polyploid hybrid with green ash (Wallander, 2008). Additionally, there is low genetic differentiation between white, velvet (*F. velutina* Torr.), and green ash (Hinsinger et al., 2013). Rapid radiation or recent exchange of genetic material could have led to these relationships (Hinsinger et al., 2013). This lack of interspecific variation is especially present in chloroplasts (Jeandroz et al., 1997). The co‐occurrence of white and green ash in all sampling locations presents the possibility of hybridization between the two species; therefore, data were analyzed as *Fraxinus* spp. due to expected low genetic differentiation. Additionally, the pairwise IBS distance analysis in PLINK did not find green and white individuals were less similar, further suggesting that our SNP data were unable to separate the two species.

Across the PCA, there was little separation based on tolerance and susceptibility categories applied by field assessment data. The exception to this pattern was the right cluster, which contained five tolerant individuals from various geographic locations in Michigan. Four of those individuals were in close proximity to the de facto introduction epicenter for *A. planipennis* (Siegert et al., 2014), indicating they have been exposed to *A. planipennis* for nearly 20 years and are still able to tolerate infestation (Marshall et al., 2013). These five trees were located in Houghton County and three Metroparks (Kensington, Oakwoods, and Willow). For this reason, outlier SNPs were identified between the four clusters on the PCA to determine which SNPs were likely causing the variation in this group.

### 4.2 SNP candidates for tolerance selection

All outlier SNPs detected between the PCA clusters had high *F_ST_* values and appeared to be responsible for the divergence of the right cluster. However, subsequent PCA on SNP variation with these outliers removed (results not shown) revealed that the five individuals in the right cluster still displayed the same pattern of divergence, indicating that these 28 loci are not the only source of variation within this group.

Throughout the outliers identified, there were similar genetic trends between trees in the middle and right clusters. The similarities between these two clusters are evident when looking at just the ten polymorphic loci that showed a pattern of almost exclusive presence in the right group. For seven of the ten loci, one to two trees from the middle cluster also had the polymorphic nucleotide. Two of these trees from the middle cluster were classified as susceptible; however, all of the trees from the middle group that had genotypic similarities with the five right group trees had no signs of *A. planipennis* attack (i.e., lacking bark spits, exit holes, woodpecker activity, and sprouting), despite being located in areas where *A. planipennis* is present.

The outlier locus 30133_137 mapped to a PTI1‐like tyrosine‐protein kinase 2. This protein is known to be involved in growth and development, as well as defense responses (Anthony et al., 2006; Floriduz et al., 2002). PTI1 serine/threonine protein kinases were described to be key components of speck disease resistance in tomatoes by amplifying signaling pathways (Sessa et al., 2000). It is possible that this gene may also play a role in defense against *A. planipennis* by amplifying pathways necessary to tolerate infestation.

From the outliers identified based on the visual PCA cluster, several were significantly associated with the tolerant group. These may be important in identifying host tolerance and susceptibility. Locus 16669_22 is another potentially important gene for host tolerance. Four outlier SNPs at this locus had distinctive patterns in individuals with either all present as the polymorphism or all in their reference form. For the five individuals in the right cluster, the reference nucleotides were retained for all four SNPs at this locus. Unfortunately, BLAST analysis did not map this locus to any known genes. Additionally, two trees that clustered in the middle PCA group also had the reference nucleotides at this locus. These two trees were classified as susceptible, but interestingly, they were the only two susceptible trees with no signs of *A. planipennis* attack. This exemplifies the coarseness of categorizing tolerance based on phenotypic characteristics of vigor and dieback. These two trees with poor vigor and high dieback may have simply displayed other manifestations of decline not associated with *A. planipennis* (e.g., Houghton County trees were along a highway and subject to salt spray).

Outliers detected between trees based on vigor rating resulted in an additional 13 loci being identified as potential candidates for host tolerance. None of these mapped to any known functional genes; however, the five outliers that did show a pattern of either the polymorphic or the reference nucleotide being present exclusively in high vigor trees are of particular interest. With five outliers associated with each of the tolerant and susceptible groups, there may be important SNPs to identify potential host tolerance and susceptibility. One constraint to mapping sequences is the amount of recombination due to divergence and availability of a representative reference genome (Sousa & Hey, 2013). Unmapped reads can also be attributed to conserved sequences across individuals (Gouin et al., 2015). Future analyses on characterizing the functionality of the outlier loci detected in this study, specifically those with associations with tolerance and susceptibility, could expose the importance of these genes and the role they may play in tolerance.

A clear link between genotypic and phenotypic data was not identified. Most likely, this was due to the coarseness of phenotype classification, which then failed to correlate with complex genetic diversity. Phenotypes were defined by tree assessments, which included categorical tree health observations and presence or absence of signs of *A. planipennis* attack. A future study may have more success if these signs are quantified at finer scales (e.g., number of exit holes per square meter, area of phloem regrowth, and location of bark splits) as opposed to whole tree values. Boyes et al. (2019) demonstrated phloem regrowth in artificially damaged trees occurred at higher rates in tolerant individuals and those without exit holes. Detailed phenotypic data would allow for more robust analyses linking genotype and phenotype, such as a mixed‐linear model. Finally, the power of genome‐wide associations is affected by the genetic complexity and heritability of a trait (Burghardt et al., 2017). As tolerance is expected to be a complex genetic trait, this increases the chance of false positive associations. To remedy this issue in future studies, as many genotypes as possible should be used along with high‐quality nucleotide data.

## 5 CONCLUSIONS

*Agrilus planipennis* has devastated populations of *Fraxinus* spp. in North America; however, the survival of some individuals despite years of exposure to *A. planipennis* is evidence of host tolerance. Understanding the mechanisms of host tolerance through genome‐wide association has the potential to restore populations with cultivars that are able to persist in the presence of *A. planipennis*. Despite the caveats presented above, this study was successful in using RAD‐sequencing in order to identify SNPs that are potential candidates for tolerance to *A. planipennis*. This was a first step toward uncovering a genetic basis for host tolerance to *A. planipennis*. Future studies are needed to identify the functionality of the outlier loci detected in this study.

## CONFLICT OF INTEREST

The authors declare no conflict of interest.

## AUTHOR CONTRIBUTION

**Cecelia E Hale:** Conceptualization (supporting); Data curation (supporting); Formal analysis (lead); Writing‐original draft (lead); Writing‐review & editing (equal). **Mark Jordan:** Formal analysis (supporting); Methodology (supporting); Writing‐review & editing (supporting). **Gloria Iriarte:** Formal analysis (supporting); Writing‐review & editing (supporting). **Kirk D Broders:** Formal analysis (supporting); Writing‐review & editing (supporting). **Andrew J Storer:** Conceptualization (equal); Writing‐review & editing (supporting). **Vamsi J Nalam:** Conceptualization (lead); Data curation (lead); Formal analysis (supporting); Methodology (supporting); Writing‐original draft (supporting); Writing‐review & editing (supporting). **Jordan M Marshall:** Conceptualization (lead); Formal analysis (supporting); Methodology (supporting); Writing‐original draft (supporting); Writing‐review & editing (lead).

## Data Availability

Data are available through NCBI https://www.ncbi.nlm.nih.gov/bioproject/PRJNA561365/ and Dryad https://doi.org/10.5061/dryad.7pvmcvdtd
